# Robot-Driven Calibration and Accuracy Assessment of Meta Quest 3 Inside-Out Tracking Using a TECHMAN TM5-900 Collaborative Robot

**DOI:** 10.3390/s26082285

**Published:** 2026-04-08

**Authors:** Josep Lopez-Xarbau, Marco Antonio Rodriguez-Fernandez, Marcos Faundez-Zanuy, Jordi Calvo-Sanz, Juan Jose Garcia-Tirado

**Affiliations:** TecnoCampus, Universitat Pompeu Fabra (UPF), 08302 Mataro, Spain; jlopezxarbau@tecnocampus.cat (J.L.-X.); mrodriguezfe@tecnocampus.cat (M.A.R.-F.); jcalvo@tecnocampus.cat (J.C.-S.); jgarciat@tecnocampus.cat (J.J.G.-T.)

**Keywords:** mixed reality, inside-out tracking, rigid spatial alignment, tracking accuracy, collaborative robot, metrology, healthcare, procrustes alignment

## Abstract

We present a systematic evaluation of the positional and rotational tracking accuracy of the Meta Quest 3 mixed-reality headset using a TECHMAN TM5-900 collaborative robot (±0.05 mm repeatability) as a highly repeatable robot-driven reference. The headset was rigidly attached to the robot’s tool flange and subjected to single-axis translational motions (200 mm along X, Y, and Z) and rotational motions (Roll ± 65°, Pitch ± 85°, and Yaw ± 85°). Each test was repeated three times, and the resulting trajectories were averaged to improve statistical robustness. Both data sources were integrated into a single Python-based application running on the same computer. The headset streamed its data via UDP, while the robot, implemented as an ROS2 node, published its data to the same host. This configuration enabled simultaneous acquisition of both streams, ensuring temporal consistency without the need for offline interpolation. All comparisons were performed in a relative reference frame, thereby avoiding the need for absolute hand–eye calibration. Coordinate-frame alignment was achieved using Singular Value Decomposition (SVD)-based rigid-body Procrustes analysis. Over 2848 synchronized samples spanning 151.46 s, the Meta Quest 3 achieved a mean translational RMSE of 0.346 mm (3D RMSE = 0.621 mm) and a mean rotational RMSE of 0.143°, with Pearson correlation coefficients greater than 0.9999 on all axes. These results show sub-millimeter positional tracking and sub-degree rotational tracking under controlled conditions, supporting the potential of the Meta Quest 3 for precision-oriented mixed-reality applications in industrial and research settings.

## 1. Introduction

In recent years, immersive virtual reality (VR) head-mounted displays (HMDs) have attracted increasing interest in physiotherapy and rehabilitation, not only as tools for therapeutic intervention but also as potential instruments for the objective assessment of human movement. Beyond their role as training interfaces, several studies have investigated the validity of integrated HMD tracking systems for quantifying clinically relevant variables such as head kinematics, postural control, and balance. From a clinical perspective, the interest in these technologies lies in their capacity to provide objective, repeatable, and low-cost measurements of functions that have traditionally been assessed using manual instruments or observational tests [1,2].

Within the musculoskeletal domain, and specifically in the cervical region, recent studies have evaluated standalone HMDs for estimating cervical range of motion (ROM) and performing sensorimotor function tests. These studies report errors comparable to clinical instruments and good intra-session repeatability, supporting the potential of HMDs for functional screening and monitoring in clinical settings [3]. In parallel, immersive VR has been explored in vestibular physiotherapy for patients with dizziness or gaze control disorders [4]. Furthermore, the integration of eye-tracking systems into modern headsets has opened new possibilities for assessing oculomotor function and vestibulo-ocular reflex (VOR) gain [5].

Despite these advances, the existing literature tends to address these functional components in a fragmented manner. Few studies have validated integrated HMD-based assessment protocols against well-characterized external references. A critical prerequisite for these clinical applications is the rigorous characterization of spatiotemporal tracking accuracy. Reproducible evaluation protocols that distinguish systematic errors from intrinsic dynamic variability are necessary for the correct interpretation of measurements [6].

Commodity VR headsets, such as the Meta Quest 3, have rapidly evolved into compact platforms that provide real-time six-degree-of-freedom (6DoF) head pose estimates through inside-out tracking. Such pose estimates are increasingly exploited as a general-purpose motion sensing modality because they are easy to deploy and do not require external cameras [7,8]. However, the achievable accuracy of inside-out tracking is not guaranteed a priori: it depends on the headset hardware, the proprietary tracking pipeline, the visual features available in the environment, and motion dynamics. For applications where millimeter-level translation errors or degree-level orientation errors may impact downstream analysis—such as the clinical assessment of cervical ROM—a rigorous calibration methodology is required. A common strategy to characterize tracking performance is to compare the HMD pose stream against an external reference sensor (e.g., optical motion capture) [9,10]. While effective, these approaches either require specialized infrastructure or introduce their own placement issues. In contrast, industrial robots and collaborative manipulators provide highly repeatable motion generation, making them an attractive reference platform for evaluating VR tracking under controlled trajectories [11]. Nevertheless, accurate validation requires rigorous spatiotemporal alignment; neglecting internal clock drift or assuming fixed delays can lead to overestimation of dynamic tracking errors [12].

Recent advances in generalized and multi-camera relative pose estimation further contextualize the present study within the broader problem of robust spatial registration. In particular, minimal and closed-form solvers for generalized camera systems have shown that reliable pose recovery can be achieved even in non-central imaging configurations and with reduced correspondence sets, including formulations based on six-point correspondences and affine correspondences [13,14,15]. Although these methods address a different estimation setting than the robot-driven headset benchmarking considered here, they are relevant because they illustrate how future validation protocols could be extended toward richer sensing configurations, multi-camera headset models, and more visually challenging environments while preserving geometric rigor.

The scope of this work is intentionally metrological: to characterize the intrinsic tracking precision of the Meta Quest 3 under controlled and reproducible conditions. Performance under dynamic, multi-axis, or visually degraded conditions is an important complementary objective, but it lies outside the scope of the present study.

Accordingly, this paper presents a robot-driven calibration and accuracy assessment of the Meta Quest 3 headset using a TECHMAN TM5-900 collaborative robot as a motion reference. Unlike studies aimed primarily at clinical or biomechanical inference [3], the goal here is to quantify how closely the 6DoF pose reported by the headset matches a repeatable reference trajectory in both translation and rotation, while providing a transparent processing pipeline that can be reproduced and adapted to similar setups. The main contribution of this work is therefore not the introduction of a fundamentally new validation paradigm, but the implementation and systematic validation of a practical, reproducible, and robot-referenced benchmarking pipeline for Meta Quest 3 tracking under controlled 6DoF trajectories.

The main contributions of this work are:

1. A practical experimental setup in which the Meta Quest 3 is rigidly mounted to the end effector of a TECHMAN TM5-900 cobot, enabling controlled and repeatable 6DoF trajectories.
2. A spatial preprocessing pipeline that rigidly aligns the robot and headset coordinate frames and ensures consistent angle handling across both pose streams.
3. A comprehensive set of translational and rotational accuracy metrics, reported per axis and in 3D, together with representative error distributions over the tested workspace and motion profiles.

The remainder of the paper is organized as follows. Section 2 describes the experimental platform, acquisition pipeline, coordinate alignment procedure, and error metrics. Section 3 presents the translational, rotational, cross-axis, and spectral results. Section 4 discusses the implications of the findings and their practical relevance. Section 5 summarizes the main limitations and outlines future research directions. Finally, Section 6 concludes the paper and provides recommendations for extending robot-based evaluation protocols for VR inside-out tracking.

## 2. Materials and Methods

### 2.1. Experimental Setup

#### 2.1.1. Hardware

TECHMAN TM5-900 Collaborative Robot (TECHMAN ROBOT, Taoyuan, Taiwan). The TM5-900 is a 6-axis collaborative robot with a 900 mm reach, 4 kg payload, and a certified repeatability of ±0.05 mm [16]. Its high repeatability makes it a reliable reference for controlled tracking evaluation.

**Figure 1:**
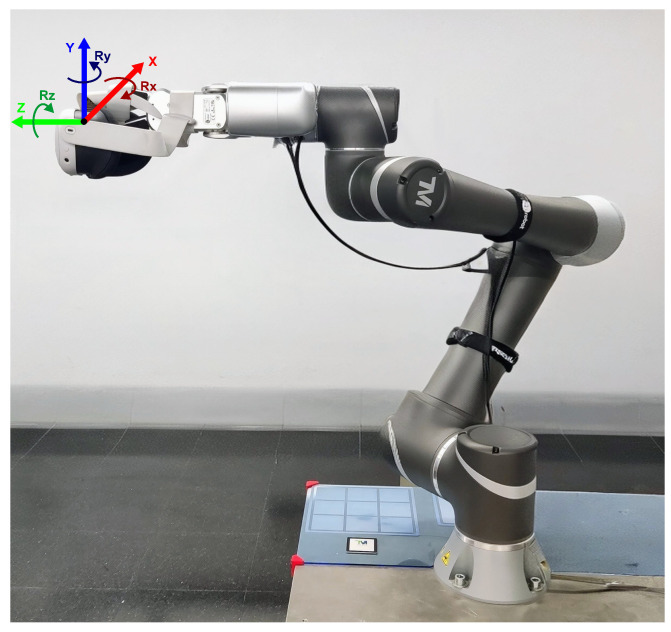
Experimental setup showing the Meta Quest 3 head-mounted display rigidly attached to the end effector of the TECHMAN TM5-900 collaborative robot via a custom 3D-printed bracket.

Meta Quest 3 HMD (Menlo Park, CA, USA). The Meta Quest 3 employs VI-SLAM tracking using six cameras (two color passthrough, two monochrome/IR hybrid, two depth) and a high-rate IMU (1000 Hz) [17]. As shown in Figure 1, the headset was rigidly mounted to the robot’s tool flange using a custom 3D-printed bracket, ensuring no relative motion between the headset and the robot’s tool-center point (TCP). Throughout all data acquisitions, the measurement conditions were kept stable: the ambient illumination was monitored with a luxmeter and maintained between 400 and 500 lux, and the visual scene (room layout and objects) remained unchanged for all trials.

#### 2.1.2. Data Acquisition

Position and orientation data were recorded synchronously from both the robot (via ROS2 (Humble) node) and the Meta Quest 3 (via a custom Unity (Version 6000.3.7f1) application streaming quaternion-based poses over UDP). Each CSV file contains:

- Timestamp *t*.
- Meta Quest 3 columns: Position ($q_{px}$, $q_{py}$, $q_{pz}$) and quaternion ($q_{qx}$, $q_{qy}$, $q_{qz}$, $q_{qw}$).
- Robot columns: Cartesian position ($r_{X}$, $r_{Y}$, $r_{Z}$) and Euler angles ($r_{Rx}$, $r_{Ry}$, $r_{Rz}$).

To improve measurement reliability, each single-axis test (three translational and three rotational) was repeated three times under identical conditions. The three recorded trajectories for each degree of freedom were then sample-wise averaged, and all subsequent alignment and error analyses were performed on these averaged trajectories. This averaging strategy reduces the influence of random per-run noise while preserving the deterministic motion profile imposed by the robot.

Both the robot (ROS2 node) and the Meta Quest 3 (Unity UDP application) stream data to the same host computer, sharing a common system clock (no inter-host synchronization is required). Both the robot and the Meta Quest 3 operate at a synchronized acquisition rate of 20 Hz: the robot exposes joint states and the Cartesian pose through the ROS2 topic interface, while the headset transmits 6DoF pose data via UDP. Timestamps are assigned at the receiving application level using the host system clock (Python (Version 3.10.12) time.perf_counter()), ensuring a shared time reference. The trials were conducted within the robot’s operational envelope, with maximum end-effector translation and rotation speeds limited to 40 mm/s and 11°/s, respectively.

#### 2.1.3. Tracking Origin and Repeatability

The coordinate system was defined relative to the virtual world origin. At the beginning of each run, the tracking origin was reset using the OpenXR SetTrackingOrigin function (configured to Floor Level) to reduce run-to-run variability.

#### 2.1.4. Robot Reference Quality and Uncertainty Considerations

In this work, the collaborative robot is used as a repeatable motion reference to generate controlled 6DoF trajectories (Figure 1). It is important to distinguish repeatability from absolute accuracy: while collaborative manipulators typically provide highly repeatable motion, the absolute end-effector pose obtained from forward kinematics can still be affected by residual kinematic parameter errors, tool-center point (TCP) definition errors, joint compliance, and mounting deflections. Consequently, the TM5-900 serves here as a controlled motion generator and reference trajectory source rather than a traceable absolute metrology instrument; the reported residuals therefore quantify agreement between the headset and the robot-driven reference, and should not be interpreted as a full decomposition of headset-only error in a traceable common frame.

Accordingly, the robot should be interpreted here primarily as a deterministic trajectory generator that enables consistent, replayable motions, rather than as a traceable metrology-grade reference. This distinction is relevant when interpreting the error metrics reported in Section 2.5. In particular, the reported error metrics intentionally include any systematic offsets that may arise from fixed mounting geometry and coordinate-frame mismatch between the robot reference and the headset tracking frame.

For completeness, the dominant contributors to the reference uncertainty in this setup are: (i) robot motion repeatability and servo variability, (ii) TCP and tool-frame definition errors (including the headset mounting geometry), (iii) compliance and micro-deflections under gravity and acceleration and (iv) numerical resolution/quantization of the robot pose stream, and (v) residual temporal misalignment.

A compact expression for the aggregated reference contribution can be written as

(1) $$u_{\mathrm{ref}}\approx \sqrt{u_{\mathrm{rep}}^{2}+u_{\mathrm{tcp}}^{2}+u_{\mathrm{comp}}^{2}+u_{\mathrm{num}}^{2}+u_{\mathrm{time}}^{2}},$$

where each term represents, respectively, repeatability, TCP/mount definition, compliance/deflection, numerical/logging resolution, and time-alignment uncertainty.

To provide a quantitative bound on the reference uncertainty, the dominant contributors were estimated as follows. The robot’s certified repeatability (±0.05 mm, 1σ) represents the most precisely characterized term [16]. TCP and mounting uncertainty, arising from the custom 3D-printed bracket, were estimated geometrically at ≤0.5 mm. Compliance-induced deflections under the applied quasi-static loading conditions (40 mm/s, 0.1 g) were estimated at <0.1 mm, consistent with values reported for similar lightweight cobots. Numerical/logging resolution contributes <0.01 mm. Temporal misalignment uncertainty is quantified in the section Temporal Synchronization and Timing Uncertainty (see below).

(2) $$u_{\mathrm{ref}}\leq \sqrt{0.05^{2}+0.5^{2}+0.1^{2}+0.01^{2}+0.2^{2}}\approx 0.55\;\mathrm{mm}$$

Combining these contributions in quadrature yields an aggregate reference uncertainty bound of approximately 0.55 mm, which should be borne in mind when interpreting the 3D RMSE of 0.621 mm reported in Section 3. Therefore, the reported results should be regarded as a benchmark of headset pose accuracy against a highly repeatable robot-driven reference. For applications requiring absolute pose agreement in a common coordinate frame, an additional spatial registration step (e.g., estimation of a constant rigid transform and, when applicable, hand–eye calibration) should be incorporated and its uncertainty reported.

##### Temporal Synchronization and Timing Uncertainty

The principal sources of residual timing uncertainty are (i) ROS2 DDS message scheduling jitter (median latency < 0.7 ms, worst case < 1 ms in loopback configurations) and (ii) UDP network stack buffering for the Meta Quest 3 stream (estimated < 5 ms on localhost). Combining these in quadrature yields a timing uncertainty of u_time ≤ 5 ms. At the nominal translational speed of 40 mm/s, this corresponds to a worst-case positional error contribution of 0.2 mm, which constitutes u_time in Equation (2).

#### 2.1.5. Reference Trajectories

The robot executed a sequence of piecewise-linear trajectories along the three Cartesian axes and along the three rotational DoFs (Roll–Pitch–Yaw). Each DoF was actuated individually with a go–and–return motion about the (0,0,0) pose: translations exhibited a peak excursion of approximately 200 mm, while orientations followed analogous ramps spanning from −65° to 65° around the X axis and from −85° to 85° around the Y and Z axes, as summarized in Table 1.

All trajectories were executed using a trapezoidal velocity profile, with a nominal translational speed of 40 mm/s and a maximum translational acceleration of 100 mm/s^2^. These motion parameters were selected to keep the dynamics within a moderate range while still exciting the headset tracking pipeline with non-negligible velocities and accelerations.

The motion commands were defined in the robot tool reference frame rigidly attached to the headset mount, with the frame origin located at the selected TCP associated with the fixture. The positive X, Y, and Z directions used for the translational tests, and the corresponding rotations about those axes, are now indicated in Figure 1 and described in the text for clarity. Translational tests were executed at 40 mm/s and a maximum translational acceleration of 100 mm/s^2^, whereas rotational tests were executed at 11°/s and a maximum rotational acceleration of 40°/s^2^. The robot was initialized from a nominal configuration selected within a regular region of the workspace, away from kinematic singularities, and the commanded trajectories were designed so that singular configurations were not approached during the experiments.

Prior to physical execution, all planned trajectories were validated in simulation using the ROS2 framework. The robot model was visualized in RViz and the motions were executed in Gazebo to verify that every target pose lay within the robot’s reachable workspace and that no joint-limit violations, singularities, or self-collisions occurred. This pre-validation step ensured that the commanded trajectories were kinematically feasible given the physical constraints of the TM5-900 (joint ranges, reach envelope, and cable routing of the mounted headset).

**Figure 2:**
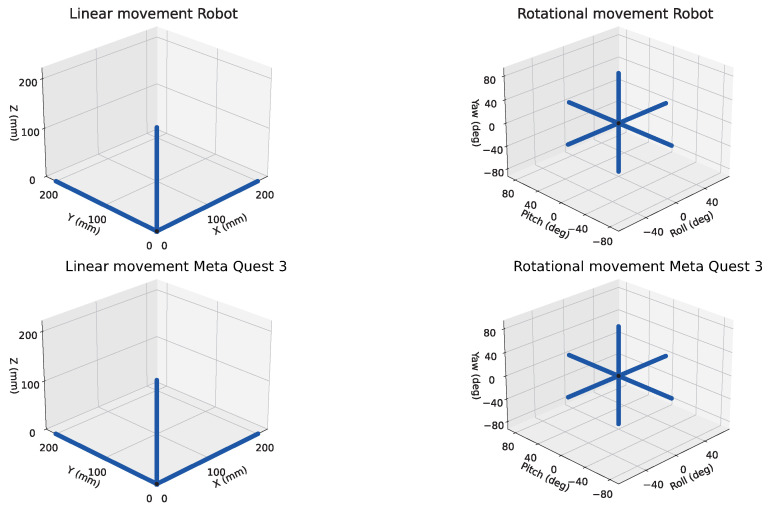
Linear (**left column**) and rotational (**right column**) 3D trajectories used for calibration. (**Top row**): reference motion commanded to the robot (end-effector position and orientation). (**Bottom row**): corresponding measurements reported by the Meta Quest 3 headset.

This forced motion enables a direct comparison between the reference robot trajectories and the positions/orientations reported by the headset under repeatable conditions (see Figure 2).

### 2.2. Relative Coordinate Framework

Let N denote the total number of synchronized samples used in the analysis. The robot and headset trajectories are represented as ordered point sets $\left\{p_{0},p_{1},\ldots ,p_{N}\right\}$ and $\left\{q_{0},q_{1},\ldots ,q_{N}\right\}$, respectively, where $p_{i}$ and $q_{i}$ denote the corresponding pose samples at index i after synchronization. The matrices Q and R denote the stacked point coordinates used in the rigid alignment step. In the error-analysis stage, $q_{i}$ denotes the headset-derived quantity at sample *i* and $r_{i}$ the corresponding robot reference value at the same sample.

To avoid the complexities of absolute hand–eye calibration, all analyses are performed in relative coordinates. Given a trajectory $p=\{p_{0},p_{1},\ldots ,p_{N}\}$, the relative displacement at sample *k* is defined as

(3) $$\Delta p_{k}=p_{k}-p_{0}$$

This removes any constant offset between coordinate frames and isolates the motion component.

### 2.3. SVD-Based Rigid-Body Alignment

The Meta Quest 3 and robot operate in different coordinate systems. To align them, we compute an optimal rigid-body transformation (rotation R* and translation t*) using SVD-based Procrustes analysis [18,19]. Given *N* corresponding point pairs ${\left\{\left(q_{i},r_{i}\right)\right\}}_{i=1}^{N}$,

1. Compute centroids:
  (4) $$\bar{q}=\frac{1}{N}\sum_{i=1}^{N}q_{i}\;\bar{r}=\frac{1}{N}\sum_{i=1}^{N}r_{i}$$
2. Cross-covariance matrix:
  (5) $$H={\left(Q-\bar{q}\right)}^{⊤}\left(R-\bar{r}\right)$$
3. Singular Value Decomposition (SVD):
  (6) $$H=U\Sigma V^{⊤}$$
4. Optimal rotation (ensuring $\det(R^{*})=+1$):
  (7) $$R^{*}=V\;\mathrm{diag}\left(1,1,\det\left(VU^{⊤}\right)\right)\;U^{⊤}$$
5. Optimal translation:
  (8) $$t^{*}=\bar{r}-R^{*}\;\bar{q}$$

The transformed Meta Quest 3 positions are then

(9) $${\hat{q}}_{i}=R^{*}q_{i}+t^{*}$$

### 2.4. Quaternion-to-Euler Conversion

The Meta Quest 3 reports orientation as unit quaternions $\left(q_{x},q_{y},q_{z},q_{w}\right)$, which are converted to Euler angles (Roll, Pitch, Yaw) using the scipy.spatial.transform.Rotation (Version 1.16.2) library with the XYZ intrinsic convention. Angle unwrapping via numpy.unwrap (Version 2.3.3) is applied to handle ±180° discontinuities, which are critical for the Yaw (Rz) tests near wrapping boundaries.

The XYZ intrinsic convention (equivalent to extrinsic ZYX, i.e., Roll → Pitch → Yaw applied sequentially to the body frame) was selected to match the Euler angle representation used by the TM5-900 robot controller, which reports orientations using the Static XYZ (sxyz) convention [16]. Consistency was verified by confirming that, for single-axis rotational tests, the reported robot Euler angle on the active axis was monotonically consistent with the headset angle after Procrustes alignment, with cross-axis angles remaining near zero.

### 2.5. Error Metrics

Four complementary metrics are used to characterize tracking accuracy:

Root Mean Square Error (RMSE):

(10) $$\mathrm{RMSE}=\sqrt{\frac{1}{N}\sum_{i=1}^{N}{\left(q_{i}-r_{i}\right)}^{2}}$$

Mean Absolute Error (MAE):

(11) $$\mathrm{MAE}=\frac{1}{N}\sum_{i=1}^{N}\left|q_{i}-r_{i}\right|$$

Maximum Absolute Error:

(12) $$\mathrm{MaxErr}=\max_{i}\left|q_{i}-r_{i}\right|$$

Pearson Correlation Coefficient (*r*):

(13) $$r=\frac{\sum_{i}\left(r_{i}-\bar{r}\right)\left(q_{i}-\bar{q}\right)}{\sqrt{\sum_{i}{\left(r_{i}-\bar{r}\right)}^{2}\sum_{i}{\left(q_{i}-\bar{q}\right)}^{2}}}$$

Percentile errors (p95, p99):

(14) $$p_{k}={\mathrm{percentile}}_{k}\;\left(\left|q_{i}-r_{i}\right|\right),\;k\in \left\{95,99\right\}$$

RMSE emphasizes larger errors (optimal for Gaussian-distributed residuals), while MAE is more robust to outliers (optimal for Laplacian distributions) [20]. MaxErr captures worst-case performance and Pearson’s *r* quantifies trajectory shape fidelity independently of scale. Percentile-based metrics $p_{95}$ and $p_{99}$—defined as the 95th and 99th percentiles of the absolute error values $|q_{i}-r_{i}|$, where $q_{i}$ is the Meta Quest 3 measurement at sample *i* and $r_{i}$ the corresponding robot reference value—complement RMSE and MAE by characterizing the upper tail of the error distribution, while remaining less sensitive than MaxErr to isolated extreme samples. The power spectral density was estimated using Welch’s method, which provides a variance-reduced spectral estimate by averaging periodograms computed over overlapping windowed segments [21].

## 3. Results

### 3.1. Translational Tracking Accuracy

Table 2 presents the per-axis and aggregate translational error metrics. The Meta Quest 3 achieved sub-millimeter RMSE on all three axes, with the Z-axis showing the lowest error (0.261 mm) and X the highest (0.478 mm). The 3D Euclidean RMSE combining all three axes was 0.621 mm.

**Figure 3:**
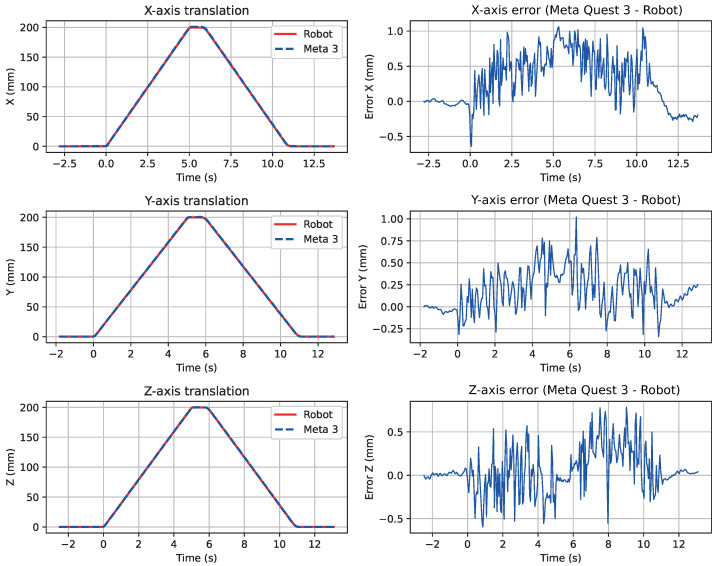
Temporal evolution and linear position error of the robot end-effector position and the Meta Quest 3 headset position along the three Cartesian axes.

Figure 3 shows the overlaid translational trajectories and the temporal evolution of translational errors on each axis. Errors remain bounded within ±1 mm throughout all tests, with no systematic drift. The robot and Meta Quest 3 traces are virtually indistinguishable, confirming the high correlation values.

**Figure 4:**
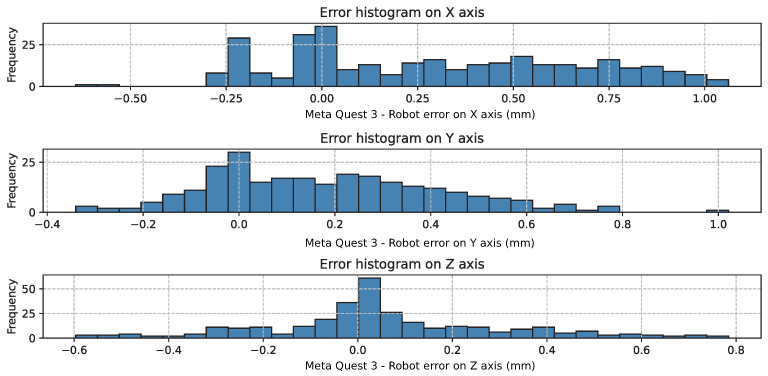
Histogram of the axis-wise translation error (in mm) after affine temporal alignment, axis mapping, and frame-rotation alignment.

Figure 4 shows the residual translational errors on each axis. The error distributions are approximately centered at zero, confirming the absence of systematic bias after the Procrustes alignment.

### 3.2. Spectral Error Analysis

**Figure 5:**
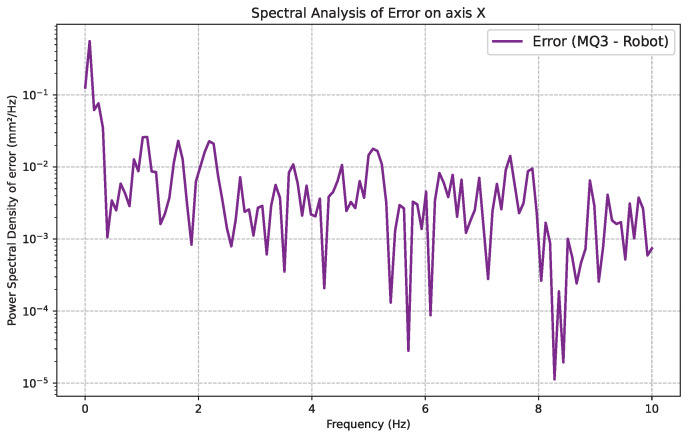

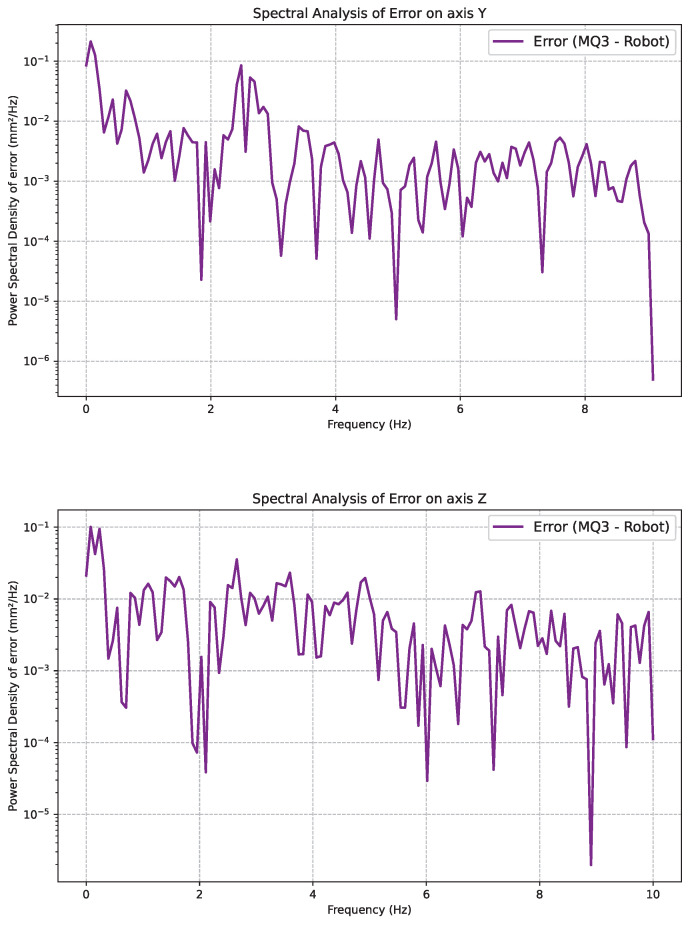
Power spectral density of the translational tracking error along the X, Y, and Z axes, estimated using Welch’s method.

To characterize the frequency content of the translational tracking residuals, the power spectral density (PSD) of the per-axis translation error was estimated using Welch’s method for the X, Y, and Z axes. The resulting spectra, shown in Figure 5, exhibit no narrowband peaks or dominant resonance lines within the analyzed 0–10 Hz range, indicating that the residual error is not driven by a single periodic disturbance or mechanical vibration.

On the X axis, the PSD shows a rapid decay from very low frequencies and then remains within a relatively narrow band over the rest of the spectrum, without pronounced tonal components. The Y axis presents a slightly more marked elevation of power at very low frequencies (below about 1 Hz), suggesting the presence of slow-varying components such as minor VI-SLAM estimation drift or quasi-static deflections in the robot–headset assembly. The Z axis PSD (Figure 5, bottom) exhibits a broadband profile broadly consistent with that of the X axis, with a moderate low-frequency elevation below 1 Hz but no identifiable resonance peaks. The relatively lower absolute power level on the Z axis compared to Y is consistent with the lower translational RMSE observed on that axis (0.261 mm vs. 0.299 mm, Table 2), confirming that the Z-tracking pipeline introduces marginally less drift or quasi-static estimation error under the tested vertical translation trajectory.

In all cases, the absolute power levels remain compatible with the sub-millimeter RMSE values reported in Table 2, supporting the interpretation that the dominant error sources behave as broadband noise with a limited low-frequency structure rather than as strong oscillatory artifacts.

### 3.3. 3D Translational Trajectories

**Figure 6:**
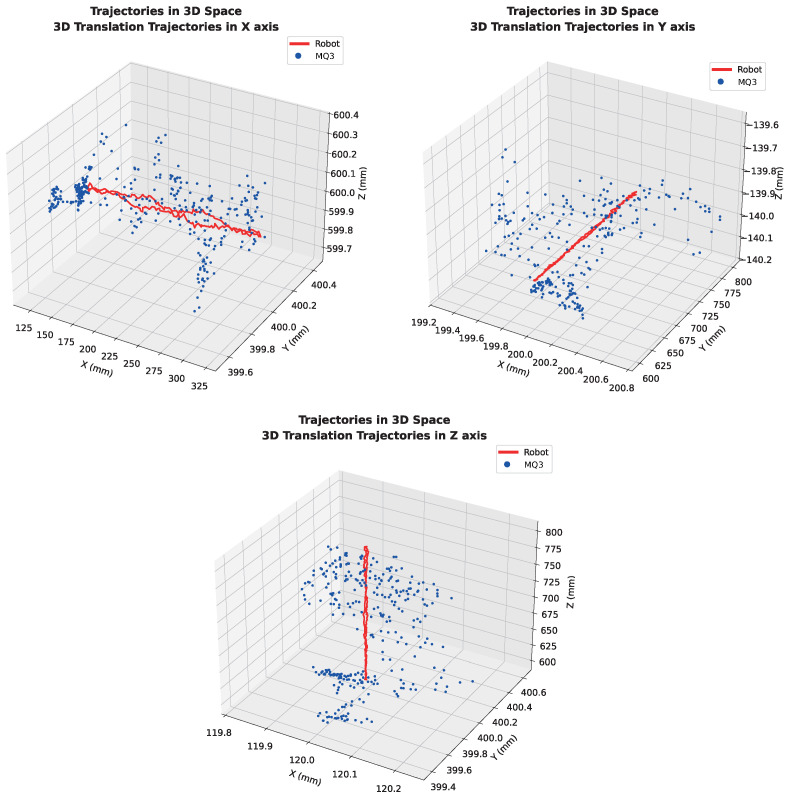
Overlaid translational trajectories (relative coordinates) for X, Y, and Z axes. Red solid: robot reference; blue dots: Meta Quest 3 aligned trajectory.

Figure 6 shows the 3D spatial trajectories for all three translational tests. The close overlap between the robot and Meta Quest 3 paths in 3D space confirms that the SVD-based alignment successfully transforms the Meta Quest 3 coordinate frame to the robot frame.

### 3.4. Rotational Tracking Accuracy

Table 3 summarizes the rotational error metrics. The Pitch axis (Ry) showed the lowest RMSE (0.058°), while Yaw (Rz) exhibited the highest (0.259°). This is consistent with the larger angular range of the Yaw test and the known sensitivity of VI-SLAM to rotations around the vertical axis.

**Figure 7:**
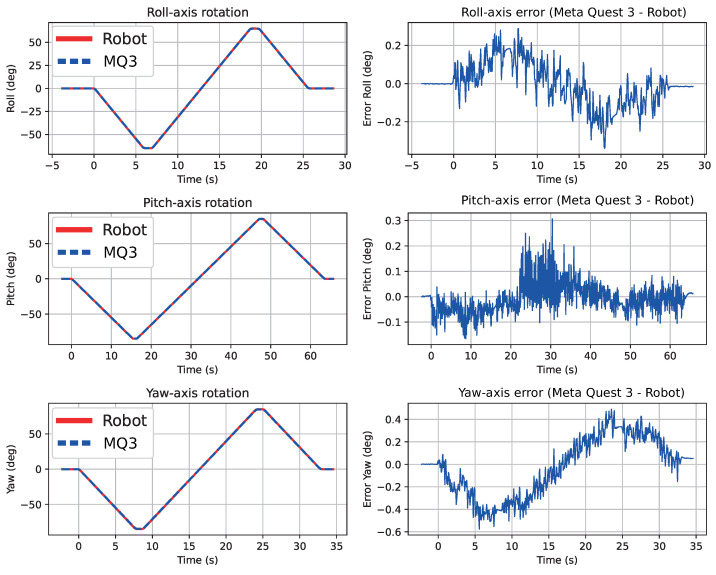
Rotational trajectories and rotational error for Roll (Rx), Pitch (Ry), and Yaw (Rz) axes.

Figure 7 shows the overlaid rotational trajectories and the temporal rotational errors for each axis. As with translation, the robot and Meta Quest 3 curves are nearly indistinguishable.

**Figure 8:**
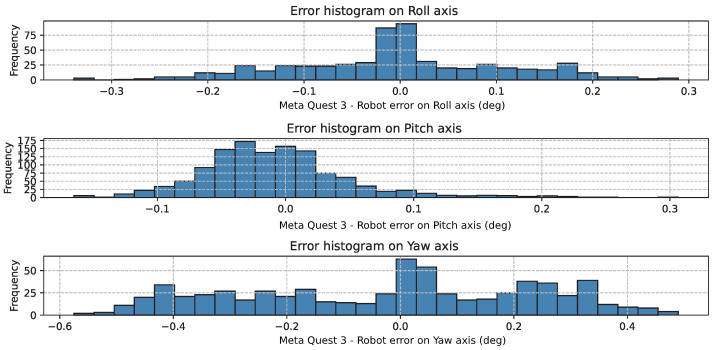
Histogram of the axis-wise rotation error.

Figure 8 shows the residual rotational errors on each axis. The distributions are approximately symmetric and centered at zero, with the Yaw axis exhibiting the widest spread, consistent with its higher RMSE.

### 3.5. Cross-Axis Coupling

Table 4 quantifies the cross-axis translational error observed during single-axis movements. During a movement along axis *i*, the RMSE on the two perpendicular axes *j* and *k* reflects unintended crosstalk. Values ranged from 0.044 mm to 0.071 mm, indicating minimal parasitic motion and confirming that the SVD-based alignment effectively decouples the three Cartesian axes.

In addition to translational crosstalk, rotational cross-axis coupling was quantified for each single-axis rotational test by computing the RMSE of the two non-commanded Euler angle residuals (Quest 3 minus robot). Specifically, during the Rx test, crosstalk was evaluated on Pitch and Yaw; during the Ry test, on Roll and Yaw; and during the Rz test, on Roll and Pitch. The resulting values, summarized in Table 5, remained small relative to the commanded angular excursion, indicating limited rotational crosstalk after frame alignment, Euler angle unwrapping, and axis normalization.

## 4. Discussion

The results show that the Meta Quest 3 can reproduce robot-driven translational trajectories with sub-millimeter accuracy and rotational trajectories with sub-degree accuracy under well-defined test conditions. Translational RMSE remained below 0.5 mm on all axes, with a 3D Euclidean RMSE of 0.621 mm, while rotational RMSE remained below 0.4° on all axes despite the relatively large angular excursions tested. The very high Pearson correlation coefficients (r>0.9999) further indicate that the headset preserves the temporal structure of the robot trajectories with no observable systematic drift or lag. Repeating each test three times and averaging the resulting trajectories also strengthens confidence in the reported values by reducing run-to-run variability.

From an application-oriented perspective, this level of tracking accuracy is relevant not only in metrological terms but also in relation to user comfort and safety. Mismatches between visual feedback and head motion, including tracking error and jitter, are well-known contributors to visually induced motion sickness (cybersickness), which can negatively affect rehabilitation adherence, particularly in vestibular populations [22].

The observed error magnitudes are also small relative to the movement ranges typically considered in cervical and vestibular assessments. Cervical range-of-motion measurements are usually on the order of several tens of degrees, and conventional clinical tools often exhibit standard errors of measurement in the range of 3° to 5° [23]. In this context, the rotational errors observed here are substantially smaller than those clinical uncertainty ranges. These findings therefore support the potential of the Meta Quest 3 as a quantitative sensing platform for controlled rehabilitation-oriented applications, although dedicated clinical validation remains necessary before broader diagnostic use can be assumed.

Table 6 places the present results in the context of representative prior studies. The translational RMSE reported here (0.346 mm) is substantially lower than the 3.19 mm reported by Banaszczyk et al. [11] for the Quest 2 using realistic head trajectories on a UR5e robot. This difference is expected. The present protocol uses slow, single-axis, piecewise-linear motions specifically designed for controlled metrological characterization, rather than naturalistic head motion. Accordingly, the present results should be interpreted as a best-case benchmark under well-defined laboratory conditions, whereas the results of Banaszczyk et al. reflect performance under more dynamic, multi-axis, and ecologically valid conditions.

Compared with optical motion-capture-based validation frameworks, the proposed protocol prioritizes reproducibility, reduced infrastructure requirements, and a unified acquisition chain. Unlike hand–eye calibration methods (AX = XB), which aim at absolute extrinsic registration between sensing and robot frames, the present study only requires rigid alignment for relative trajectory comparison after removal of constant offsets [24]. For this reason, an SVD-based Procrustes solution was adopted, as it provides a closed-form least-squares estimate with well-established numerical stability for corresponding 3D point sets [25,26,27]. By moving both streams to a common origin before alignment, the number of calibration unknowns is reduced while preserving a rigorous comparison framework. This choice is also consistent with previous sensor-alignment literature [18,19].

An important practical aspect of the present work is the tightly integrated acquisition pipeline, in which both the collaborative robot and the Meta Quest 3 stream their poses to a single Python application running on the same computer. This design reduces the need for offline clock-drift estimation and temporal interpolation, thereby addressing a source of uncertainty that is often underemphasized in previous studies. Combined with the relative coordinate framework and rigid-body Procrustes alignment, this architecture makes the methodology easier to reproduce while still enabling a rigorous comparison of translational and rotational tracking performance.

The observed anisotropy in rotational accuracy, with larger errors around the vertical Yaw axis, is also consistent with the known sensitivity of visual–inertial SLAM pipelines to rotations that reduce the effective parallax of visual features [28]. Even in this more challenging configuration, however, the Meta Quest 3 remained within a sub-degree error range and maintained excellent correlation with the robot reference. Likewise, cross-axis translational coupling remained below 0.08 mm RMSE during nominally single-axis motions, indicating negligible parasitic motion and limited crosstalk for the trajectories considered.

The spectral analysis of the translational residuals supports the same interpretation. The estimated power spectral densities do not show dominant periodic components, suggesting that the residual discrepancies between the robot and the headset are not driven by strong oscillatory modes or mechanical resonances. Instead, the error behaves predominantly as low-amplitude broadband noise. The slightly elevated low-frequency content observed on the Y and Z axes suggests the presence of slow-varying estimation effects within the Meta Quest 3 VI-SLAM pipeline, but these variations remain well within the sub-millimeter error envelope measured in this study.

## 5. Limitations

While the proposed robot-driven evaluation protocol provides a controlled and highly repeatable environment to characterize the Meta Quest 3 tracking performance, several limitations must be acknowledged.

First, the TECHMAN TM5-900 collaborative robot is used as a repeatable trajectory generator rather than as a fully traceable metrology-grade reference. Consequently, the reported error statistics include robot-related uncertainty contributions (e.g., kinematic parameter errors, TCP definition, compliance, and mounting effects) already discussed in Section 2.1.4, which are not independently identified or compensated in the present work.

Second, all analyses are performed in a relative coordinate framework, with errors computed after rigid Procrustes alignment between the robot and headset pose streams. This design deliberately avoids the need for absolute hand–eye calibration and makes the protocol easier to reproduce, but it also means that constant spatial offsets and residual frame misregistration are not explicitly quantified, so the metrics primarily reflect repeatability rather than absolute pose agreement.

Third, the experimental protocol focuses on single-axis, piecewise-linear trajectories with moderate velocities and accelerations under controlled setup. While the spectral analysis suggests that residual errors are mainly broadband in nature, the observed sub-millimeter translational and sub-degree rotational errors still characterize a best-case scenario. Performance in more demanding settings—such as highly dynamic motions, complex multi-axis paths, cluttered environments, or degraded visual texture and lighting—remains outside the scope of this study and may exhibit larger errors.

Fourth, the evaluation considers a single headset unit, a single robot platform, and a specific mechanical mounting configuration. Device-to-device variability, alternative mounting geometries, and other robot types are not explored. In particular, the custom 3D-printed bracket is assumed to be rigid, but small unmodeled flex or play could contribute to the overall error budget.

Fifth, because the experiments were conducted in a stable laboratory scene with controlled illumination and static visual features, the reported metrics should be interpreted as a reproducible benchmark under well-defined operating conditions. Further experiments in visually degraded, cluttered, or dynamic environments would help assess the robustness of the tracking pipeline under a broader range of application scenarios.

Finally, the processing pipeline relies on a unified acquisition architecture in which both the robot and the headset stream data to the same computer. This design reduces temporal misalignment compared to independently logged streams, but residual timing uncertainty due to communication delays, buffering, and software scheduling cannot be fully excluded and is not separately quantified in the reported metrics.

## 6. Conclusions

This work presented a robot-driven protocol for quantifying the 6DoF tracking accuracy of the Meta Quest 3 mixed-reality headset using a TECHMAN TM5-900 collaborative robot as a highly repeatable motion reference. By rigidly mounting the headset to the robot, streaming both pose sources into a unified acquisition pipeline, and aligning their coordinate frames through SVD-based rigid-body Procrustes analysis, the proposed methodology enables a transparent and reproducible benchmark under controlled conditions. To improve statistical reliability, all trajectories were pre-validated in simulation and repeated multiple times.

Under the tested single-axis trajectories, the Meta Quest 3 achieved a 3D Euclidean translational RMSE of 0.621 mm and a mean rotational RMSE of 0.143°, with Pearson correlation coefficients greater than 0.9999. Cross-axis translational coupling remained negligible (<mm), and no systematic drift was observed during the tests. These results indicate that, relative to the highly repeatable robot-driven reference used here, the Meta Quest 3 provides a level of tracking precision that is promising for applications requiring accurate pose estimation under controlled laboratory conditions.

The present findings establish a reproducible benchmark for the tested setup, but they should be interpreted within the scope of the experimental design. The results were obtained using a single headset unit, one robot platform, one mounting configuration, and stable environmental conditions. Moreover, the study was intentionally designed around relative-frame comparison rather than absolute hand–eye calibration. For this reason, application-specific validation remains necessary before the device can be adopted in clinical or industrial workflows with defined operational tolerances.

Beyond the numerical results themselves, the proposed pipeline provides a practical framework for future headset benchmarking. Design choices such as relative-frame analysis and rigid spatial alignment without full absolute registration simplify the metrological evaluation of mixed-reality devices while preserving methodological rigor.

Several directions for future work follow naturally from the present study. First, evaluating multiple Meta Quest 3 units under different lighting conditions and levels of visual feature richness would help characterize inter-device variability and the robustness of the tracking pipeline. Second, more complex, high-acceleration, and multi-axis trajectories should be introduced to better approximate dynamic human motion. Third, the use of an external metrology-grade reference, such as an optical motion-capture system, would enable a more complete uncertainty analysis and help separate the error contributions of the robot, the headset, and the alignment process. For applications requiring common-frame agreement, future studies should also include explicit absolute-registration experiments, including estimation of the constant rigid transform between systems and, where appropriate, hand–eye calibration. In addition, the mechanical contribution of the mounting interface should be characterized in greater detail by comparing alternative mounting solutions and quantifying flex- or play-induced errors. Finally, dedicated timing experiments should be carried out to isolate the contributions of communication delay, buffering, and software scheduling within the unified acquisition architecture.

Additionally, although the present protocol deliberately avoids absolute hand–eye calibration by operating in a relative coordinate framework, future work should quantify the constant spatial offset between the robot TCP frame and the headset tracking frame, for example through a dedicated AXYB-type hand–eye calibration procedure [6], in order to extend the protocol to applications requiring absolute pose agreement. Residual temporal uncertainty, discussed in the section, Temporal Synchronization and Timing Uncertainty, could also be reduced in future implementations through hardware-level timestamp injection or PTP-based clock synchronization between the HMD streaming interface and the robot controller.

## Figures and Tables

**Table 1:** Summary of experimental tests.

Test	Range	Samples	Duration (s)
Translation X	200 mm	330	16.45
Translation Y	200 mm	331	16.50
Translation Z	200 mm	312	15.55
Rotation Rx (Roll)	±65°	590	32.39
Rotation Ry (Pitch)	±85°	615	33.77
Rotation Rz (Yaw)	±85°	670	36.82
Total	-	2848	151.46

**Table 2:** Translational tracking error metrics.

Translation (mm)						
Axis	RMSE	MAE	$p_{95}$	$p_{99}$	MaxErr	Pearson r
X	0.478	0.374	0.912	1.013	1.062	0.999994
Y	0.299	0.229	0.596	0.761	1.021	0.999996
Z	0.261	0.185	0.555	0.719	0.784	0.999994
Mean	0.346	0.263	0.688	0.831	0.956	0.999993

**Table 3:** Rotational tracking error metrics.

Rotation (°)						
Axis	RMSE	MAE	$p_{95}$	$p_{99}$	MaxErr	Pearson r
Roll (Rx)	0.111	0.083	0.211	0.281	0.339	0.999999
Pitch (Ry)	0.058	0.044	0.114	0.184	0.306	0.999999
Yaw (Rz)	0.259	0.217	0.443	0.496	0.575	0.999999
Mean	0.143	0.115	0.256	0.320	0.407	0.999999

**Table 4:** Cross-axis translational RMSE (mm) during single-axis tests.

Active Axis	Cross X (mm)	Cross Y (mm)	Cross Z (mm)
X test	-	0.044	0.071
Y test	0.052	-	0.062
Z test	0.052	0.052	-

**Table 5:** Cross-axis rotational RMSE (°) during single-axis tests.

Active Axis	Cross Roll (°)	Cross Pitch (°)	Cross Yaw (°)
Roll test (Rx)	-	0.081	0.041
Pitch test (Ry)	0.181	-	0.137
Yaw test (Rz)	0.204	0.061	-

**Table 6:** Quantitative comparison with representative prior evaluations of VR/MR headset 6DoF tracking accuracy.

Study	Headset	Reference	RMSE	Methodology
Present work	Meta Quest 3	TM5-900 cobot	0.346 mm 0.143°	Single-axis, SVD–Procrustes, relative frame
Banaszczyk et al. [11]	Quest 2 Quest Pro	UR5e + OptiTrack	3.19–5.29 mm ^a^	Naturalistic head trajectories
Niehorster et al. [9]	HTC Vive	Optical mocap	0.08 mm 0.1°	Controlled static dynamic poses
Trinidad-Fernández et al. [3]	Meta Quest 2	Clinical goniometry	2.3–5.4° ^b^	Clinical ROM assessment

^a^ Reported across multiple headset units and trajectory types. ^b^ Reported as standard error of measurement for cervical ROM; not directly comparable to controlled-lab RMSE.

## Data Availability

The raw data supporting the conclusions of this article will be made available by the authors on request.
